# Multichannel direct transmissions of near-field information

**DOI:** 10.1038/s41377-019-0169-3

**Published:** 2019-07-03

**Authors:** Xiang Wan, Qian Zhang, Tian Yi Chen, Lei Zhang, Wei Xu, He Huang, Chao Kun Xiao, Qiang Xiao, Tie Jun Cui

**Affiliations:** 1 The State Key Laboratory of Millimeter Waves, Southeast University, Nanjing, 210096 China; 2 National Mobile Communications Research Lab, Southeast University, Nanjing, 210096 China

**Keywords:** Photonic devices, Metamaterials

## Abstract

A digital-coding programmable metasurface (DCPM) is a type of functional system that is composed of subwavelength-scale digital coding elements with opposite phase responses. By configuring the digital coding elements, a DCPM can construct dynamic near-field image patterns in which the intensity of each pixel of the image can be dynamically and independently modulated. Thus, a DCPM can perform both spatial and temporal modulations. Here, this advantage is used to realize multichannel direct transmissions of near-field information. Three points are selected in the near-field region to form three independent channels. By applying various digital phase codes on the DCPM, independent binary digital symbols defined by amplitude codes (namely, weak and strong amplitudes) are transmitted through the three channels. The measured near-field distributions and temporal transmissions of the system agree with numerical calculations. Compared with the conventional multichannel transmission, the proposed mechanism achieves simultaneous spatial and temporal modulations by treating DCPM as an energy radiator and information modulator, thereby enduing DCPM with high potential in near-field information processing and communications.

## Optics: Metasurfaces take control in the quest to manipulate light

Scientists have developed a programmable metasurface capable of controlling both the spatial and temporal modulations of light, opening the door for tunable optical components that are flat and easily integrated into other devices. Metasurfaces are ultrathin materials made from metals or semiconductors that can modulate the amplitude, phase, and polarization of light waves. However, most metasurfaces currently available are static in nature, with their optical properties fixed during fabrication. Now, Xiang Wan and colleagues from Southeast University in China have developed a digital-coding programmable metasurface (DCPM) that can dynamically control light in real time. Made from subwavelength-scale digital coding elements with opposite phase responses, the proposed DCPM is capable of multi-channel direct transmissions of near-field information and could be used in a range of applications, including wearable devices, autonomous vehicles, and sensing, imaging, and display technologies.

## Introduction

With the rapid growth in the number of devices that are in use, the fifth generation (5G) of communication will involve communications not only between humans but also between objects and between humans and objects. Hence, in addition to improving the network capacity and the spectrum efficiency, establishing additional communication modes is an evolution direction of 5G. Device-to-device (D2D) communication is a type of communication technology that can directly transmit and receive information between terminals; hence, it can reduce the burden on the base stations and decrease the end-to-end delay, improve the spectrum efficiency and reduce the transmission power of the terminals. However, the communication systems that are based on the classical superheterodyne architecture can no longer satisfy the requirements of modern communications due to the high complexities, high cost, and large volumes. Furthermore, the digitization of modern communication systems has evolved from baseband to radio frequency links and antennas. Therefore, more flexible hardware architectures, new types of information, and innovative breakthroughs in the theories of information and communication are urgently needed for satisfying the substantial demands of 5G for “huge capacity, huge connections, and extensive applications”.

Metamaterial^1,2^, which is a type of artificial material, has been demonstrated to control macroscopic phenomena of electromagnetic (EM) waves, such as negative reflections^3,4^, perfect lenses^5^, and invisible cloaks^6–10^. Microscopic phenomena, such as molecular spontaneous emission^11^ and bound states in the continuum^12^, can also be manipulated by metamaterials. After years of development, metamaterials have been fully developed in various morphologies, from acoustic waves^13^ to light^14^. A metasurface is a two-dimensional morphology of metamaterials. According to a generalized version of Snell’s law^15^, the manipulation of EM waves does not necessarily require bulky gradient metamaterials, which typically have a size of several wavelengths according to effective medium theory^16^; instead, the manipulations can be realized within the subwavelength scale. Many types of metasurfaces have been proposed for controlling the phases^17–19^, amplitudes^20-23^, and polarizations^24–29^ of EM wave fronts and for novel applications such as holographic imaging^30–32^, vortex beams^33–35^ and conversions of propagation waves, and surface waves^36,37^. Active metasurfaces have also been proposed for manipulating the EM waves in various frequency regimes^38–41^.

With further research, digital coding metasurfaces (DCMs)^42–45^ were proposed for designing more functional devices and/or systems, thereby introducing metasurfaces into informatic fields^46,47^, in which the digital coding metasurface can manipulate EM waves by arranging the states of digital elements instead of designing effective medium parameters. Moreover, digital coding programmable metasurfaces (DCPMs) were proposed for performing dynamic spatial modulations^48–50^. A DCPM is a type of artificial encoded aperture that is constructed of digital elements and digital coding information is written into the aperture by independently applying a driven voltage on each digital element. Hence, a DCPM can control EM waves dynamically in a programmable way. However, only far-field EM features (e.g., radiation and scattering patterns) of DCMs and DCPMs have been explored in the literature^42–50^. According to Fresnel diffraction theory, the aperture field is related to the diffracted field via the Fourier transform. Therefore, spatial modulations of near fields can be realized by configuring the digital coding schemes of DCPMs, which have higher information capacities.

Since the aperture codes of a DCPM change over time, the digital coding mechanism naturally involves temporal operations. Hence, for each position on the near-field plane, the amplitudes and phases of near fields can be modulated in the time domain by sequentially changing the aperture codes. Therefore, DCPMs can perform near-field modulations in both the space and time domains, which can be used to directly transmit near-field information through multiple channels. To interpret the mechanism, a near-field communication system with multiple channels is established using a DCPM. Three points are selected in the near-field region to form three independent channels. By applying space-time digital codes on a DCPM, independent binary symbols are transmitted through the three channels. The measured near-field distributions and temporal transmissions of the system accord with the numerical calculation results. As the spatial and temporal modulations are directly realized by a DCPM, the presented mechanism of multichannel transmission of near-field information will enrich the modes of 5G communication and improve the mechanisms of near-field information processing and communications.

## Results

### Near-field patterns that correspond to various coding schemes

**Fig. 1:**
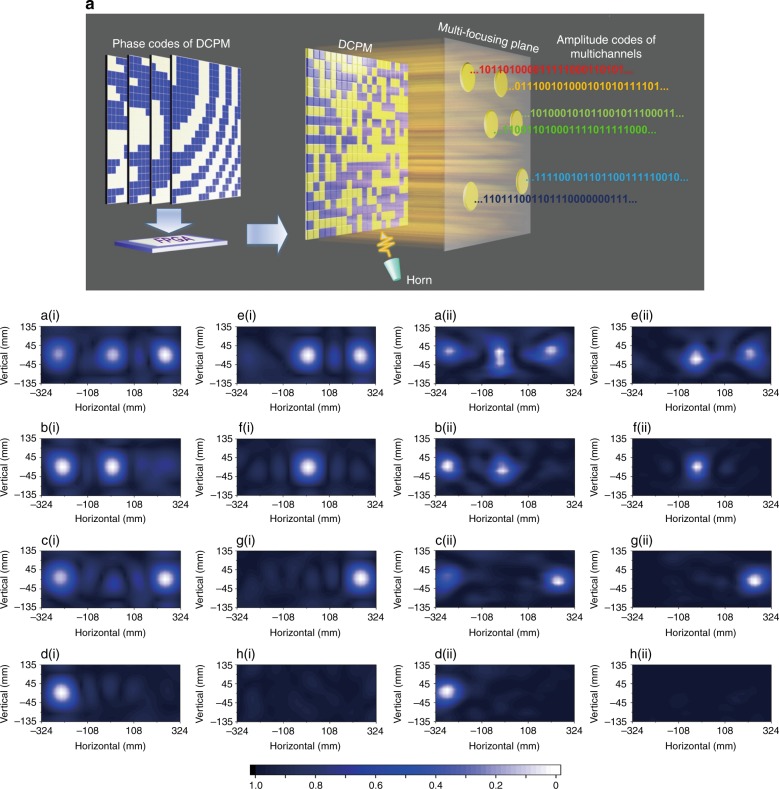
Diagram of the multichannel transmission system and near-field patterns of various combinations of symbols in the three channels. **a** The coding schemes are stored in a field-programmable gate array (FPGA) in advance and are recalled to sequentially configure DCPM so that the near field will be modulated in the space and time domains. As a result, distinct information can be transmitted through each channel. **a(i)**–**h(i)** The simulated results. **a(ii)**–**h(ii)** The measured results

A conceptual diagram of the multichannel transmission system is presented in Fig. 1a. The presented DCPM consists of 400 elements. Figure S4 presents the detailed structure of an element and the simulated scattering coefficients. Each element can render either of two types of states, which possess opposite reflection phases and similar reflection amplitudes. Hence, the aperture configurations of a DCPM can be described by a matrix of binary phase codes. By applying specially designed phase codes, a DCPM can focus the EM fields at arbitrary specified points and the field intensities at these points can be controlled independently. If one denotes the strong field intensity as digital symbol “1” and the weak field intensity as digital symbol “0”, these points will function as multiple transmission channels of binary symbols. To continuously transmit the binary digital symbols, the DCPM can be configured sequentially via a series of coding schemes, which are stored in a field-programmable gate array (FPGA) in advance. As a result, different binary symbols can be transmitted at different near-field points, thereby realizing multichannel direct transmission of near-field information.

By combining spatial and temporal modulations, a multichannel transmission system can be realized by using a DCPM. Photos of the fabricated DCPM are shown in Fig. S1. The near-field patterns of the system are presented in the lower part of Fig. 1, in which each pattern has been normalized to its maximum value. The left two columns are calculated results and the right two columns are measured results. The intensities of three points are modulated independently by changing the coding schemes of the DCPM. If high intensity represents symbol “1” and lower intensity symbol “0”, then the three points form three transmission channels and binary digital symbols can be transmitted through these channels.

Because the presented multiple channels are realized by rearranging the near-field energies at three channels, the amplitudes of the symbols may fluctuate in the space and time domains. In the space domain, the imbalanced energy of the channels is caused by factors such as the asymmetry of the coding scheme and the fabrication errors. The symbol “1” may differ in amplitude among the three channels due to this imbalanced energy. In the time domain, there is an inherent imbalance among the channels. If only one channel transmits the symbol “1”, the signal intensity of this channel is stronger than that of each channel if all three channels transmit the symbol “1”. Because the energy is focused on a single channel rather than being distributed among three channels, the amplitude of a symbol in the same channel will depend on the combination of symbols in the three channels.

### Channel strengths and transmission efficiencies

**Fig. 2:**
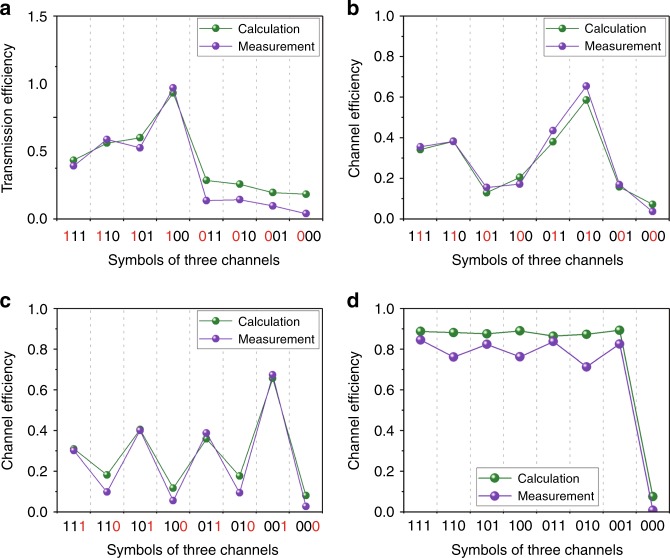
Channel strengths and transmission efficiencies. **a** The channel efficiency of channel 1. **b** The channel efficiency of channel 2. **c** The channel efficiency of channel 3. **d** The transmission efficiency. The horizontal ordinate in each figure represents combinations of binary symbols in the three channels. The red digits represent the transmitted symbols in the corresponding channel

To evaluate the fluctuations of the channel strengths, the near fields are integrated around the focal point within an integral area, in which the field values are higher than 10% of the value at the focal point. All the channel strengths are normalized to a fixed value, which is obtained by integrating the values of near-field pattern “010”. Figure 2a–c presents the normalized channel strengths. If only one channel transmits symbol “1”, the channel strength is stronger compared to the cases in which two or three channels transmit symbol “1”. Hence, the number of channels affects the strengths of the channels. As the independent channels are established by focusing the near-field energy, the channel strengths can be increased by enlarging the aperture of the DCPM. The squares of the sums of the channel strengths are defined as the transmission efficiencies in Fig. 2d. The transmission efficiencies are unaffected by the combinations of symbols in the three channels; hence, increasing or reducing the number of channels will not affect the total energy that is transmitted by the DCPM. To analyze the effect of multiple channels on the receiving performance, Fig. S2 compares various numbers of channels in terms of the bit error rate (BER). The results in Fig. S2 demonstrate that the multiple channels that are produced by DCPMs can contribute to the reduction of BER.

### Multichannel transmission of symbols

**Fig. 3:**
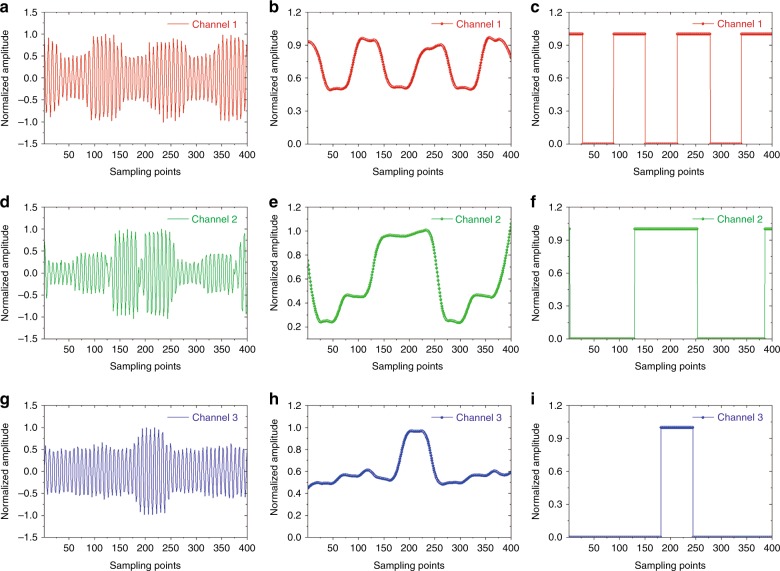
Waveforms of the measured signals. **a**, **d**, **g** The measured waveforms in the three channels. **b**, **e**, **h** The waveforms after Hilbert transformations. **c**, **f**, **i** The waveforms after threshold decisions

The results of the following transmission experiments demonstrate that the DCPM can transmit different near-field symbols among channels. To receive the symbols, a general signal processing board was used to sample the signal at each channel. For convenience of sampling, the carrier waves of the transmitted signals were converted from 10 GHz to 3 MHz. The period of each symbol is 2 μs and the sampling frequency is 31.44 MHz. Hence, each period of the signal will be sampled by 63 times. The digital receiver can record 400 sampling points. The left column of Fig. 3 presents the sampled signals in three independent channels. However, after Hilbert transformations (the middle column of Fig. 3) and threshold decisions (the right column of Fig. 3), the extracted envelopes of the signals demonstrate that the sampled signals are exactly the signals that were transmitted by the DCPM; therefore, the DCPM can perform both spatial and temporal modulations on the near fields. Hence, multichannel direct transmissions of near-field information are realized using the DCPM.

## Discussion

For spatial modulations, the fabricated DCPM was measured in a near-field microwave anechoic chamber. Figure S1 presents a photograph of the measuring environment. In the procedure of measuring the near fields, the DCPM was placed on a wood platform and a waveguide probe was positioned 600 mm away from the DCPM to record the near-field pattern with a step size of 0.018 mm. All measurements were automatically controlled by the software system of the anechoic chamber. For convenience of measurement, the aperture codes of the DCPM were held constant during each measurement. The centers of the measuring plane and the DCPM were aligned for all measurements. The three channels were selected to be horizontally distributed to alleviate the shielding effects. For temporal modulations, the DCPM was configured sequentially via a series of coding schemes and a general signal processing board was used as a digital receiver to sample the signals that were recorded by the waveguide probe. Figure S2 presents a schematic diagram of the multichannel transmission process. The waveforms of the signals were recorded by the software of the general signal processing board, as presented in Fig. 3.

From both calculations and measurements, we observe the fluctuations in the space and time domains. However, they are not sufficiently violent for hindering the identification of symbols. Typically, the receivers of communication systems have sufficient dynamic ranges for covering the fluctuations. In the presented experiments, the period of the transmitted symbols is selected as 2 μs for convenience of sampling. The minimum period of the transmitted symbols is determined by the fastest switching time of the DCPM. The main limiting factor of the speed of the DCPM is the switching time of the Positive-Intrinsic-Negative (PIN) diode and the DC feeding method. In the present work, all units of the DCPM are controlled by a digital circuit board (e.g., FPGA) in parallel; hence, the switching time of the whole aperture is very close to the switching time of a single PIN diode. The fastest switching time of the DCPM is 25 ns according to the test. However, short periods may result in larger errors in the transmitted symbols. Hence, the period of the transmitted symbols must balance the rate and accuracy of the symbols.

In conclusion, the dynamic property of DCPMs is used to realize multichannel direct transmission of near-field information. Spatial modulations are performed on the near fields to form three transmission channels and independent binary amplitude modulations are performed on each channel to transmit distinct digital information. Both spatial and temporal modulations are achieved by directly configuring a DCPM. Comparing with the conventional multichannel transmissions, the new transmission mechanism does not require phase shifters for the spatial modulations or baseband modulators for the temporal modulations. These advantages endow DCPMs with high potential in near-field information processing and high-capacity communications.

## Materials and methods

### Spatial modulations

**Fig. 4:**
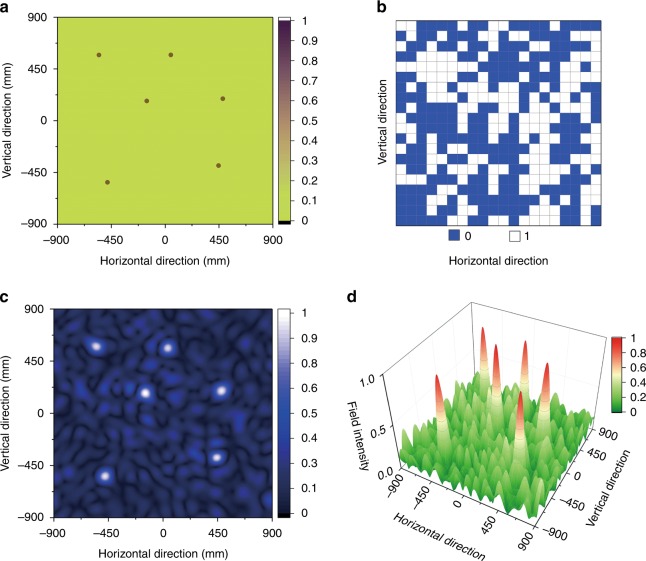
Example of spatial modulation. **a** The specified six focal points. **b** The corresponding matrix of the binary phase code of DCPM. **c** The near-field pattern was calculated from the phase code. **d** The three-dimensional near-field pattern that was calculated from the phase code

First, the capacity of a DCPM for performing near-field spatial modulations is investigated. As an example, six focal points are defined arbitrarily on a near-field plane that is 600 mm away from the DCPM. Figure 4 shows the specified points and the corresponding binary phase code matrix of the DCPM. According to the near-field pattern, six focal points appear at the specified positions. In addition to the focal points, other complex near-field patterns can be realized by configuring the DCPM. In Fig. S5, the near-field pattern is defined as the shape of the letter “T”. Both the calculations and measurements show a letter “T” on the near-field plane, thereby demonstrating that DCPM can distribute near-field patterns in an arbitrary way. For all the near-field patterns, the derivations of the matrix of binary phase codes are detailed in Fig. S6.

**Fig. 5:**
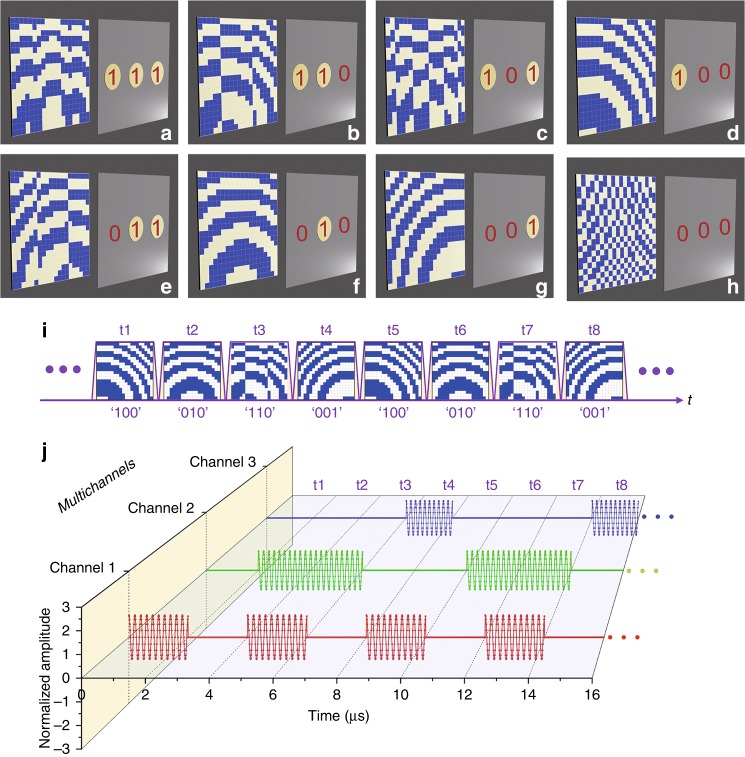
Spatial modulations and temporal modulations that are based on a DCPM. **a**–**h** The transmitted symbols of three channels and the corresponding binary phase codes. **i** The timing sequences of the coding schemes of a DCPM. **j** The waveforms in the three channels

Based on the spatial modulations, multiple channels for the near-field transmission can be physically established. For convenience of testing, three points are specified as three transmission channels. When the DCPM focus the energy at one point, the digital symbol “1” will be transmitted through the corresponding channel; otherwise, the digital symbol “0” will be transmitted. To independently transmit binary digital symbols through the three channels, eight types of coding schemes for the DCPM are needed. The correspondences between the coding schemes and the transmitted symbols in the three channels are presented in Fig. 5a–h.

### Temporal modulations

For temporal modulations, one can specify a series of coding schemes for the DCPM for transmitting a series of binary symbols. As an example of independent temporal modulations, periodic bytes that each contain four binary symbols are specified in the three channels: “1010”, “0110”, and “0001” in channel 1, channel 2, and channel 3, respectively. Figure 5i shows the timing sequence of the coding schemes that will be used to configure the DCPM for the transmission of the specified binary symbols of each channel. Figure 5j presents the corresponding signal waveforms of the three channels. The carrier wave is defined at 10 GHz; hence, the signals will be amplitude-modulated sinusoidal waves. Because each coding scheme of the DCPM remains unchanged for 2 μs, the transmitted symbol will also last for 2 μs. For the transmitted signals, high amplitude represents symbol “1”, while low amplitude represents symbol “0”. The envelopes of the signals in the three channels correspond to the transmitted binary symbols.

## Supplementary information

Multichannel direct transmission of near-field information_SM
