# Supplementary material for: Multichannel direct transmissions of near-field information
Source: Light Sci Appl. 2019 Jul 3;8:60. doi: 10.1038/s41377-019-0169-3 (PMC6804601; doi:10.1038/s41377-019-0169-3)
Supplement: Supplementary file 1 — Multichannel direct transmission of near-field information_SM [file 41377_2019_169_MOESM1_ESM.docx]

**Supplementary information for**

**Multichannel direct transmissions of near-field information**

Xiang Wan^1^, Qian Zhang^1^, Tian Yi Chen^1^, Lei Zhang^1^, Wei Xu^2^, He Huang^2^, Chao Kun Xiao^2^, Qiang Xiao^1^, and Tie Jun Cui^1^*

^1^The State Key Laboratory of Millimeter Waves, Southeast University, Nanjing 210096, China

^2^National Mobile Communications Research Lab, Southeast University, Nanjing 210096, China

*Email: tjcui@seu.edu.cn

**Supplementary Note 1: Photos of the fabricated DCPM**


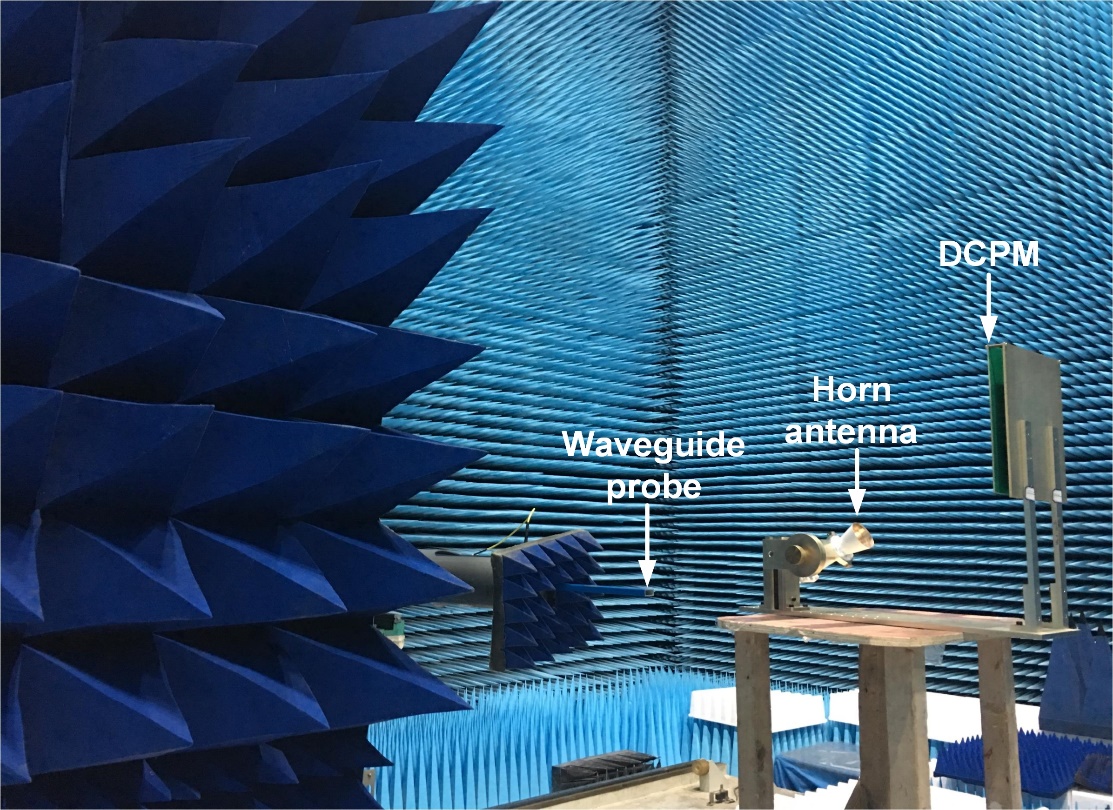


**Fig. S1 Photographs of the fabricated DCPM.**

The near-field patters of the DCPM were measured in a microwave anechoic chamber. A horn antenna was used to illuminate DCPM with continuity single-frequency wave (10GHz), and a waveguide probe in front of the DCPM was used to record the values by a step of 0.018mm. Because the horn antenna and the trestles will affect the near fields, the transmission channels were chosen to be distributed horizontally to avoid the interferences.

**Supplementary Note 2: Bit error rate of multiple channels**

The multiple channels produced by DCPM can be used to transmit different signals in different channels. Also, it can be used to reduce the bit error rate if the signals are transmitted simultaneously through multiple channels. To interpret this performance, the BER for the cases of “001”, “011” and “111” are calculated in Fig. S2. As indicated by Fig. 2, the total transmitted energy in the three cases are almost the same with each other. The calculations are based on the channel strengths given in Fig. 2, and the channels are assumed to be additive white Gaussian noise (AWGN) channels. For binary amplitude modulations, the theoretical minimum BER is calculated as $e^{-{SNR}/4}/2$ in the case of non-coherent demodulation. From the calculations, it is found that the BER is reduced and gradually tends to the theoretically values.


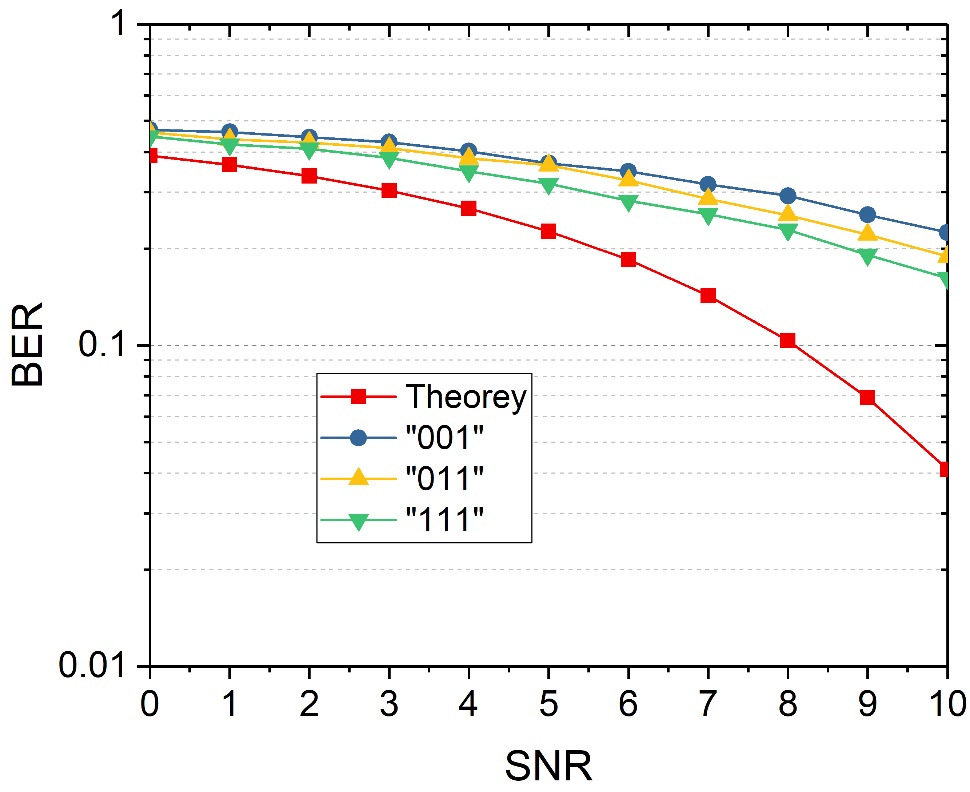


**Fig. S2 BER for the cases of different number of channels.**

**Supplementary Note 3: Schematic diagram of the multi-channel transmission**


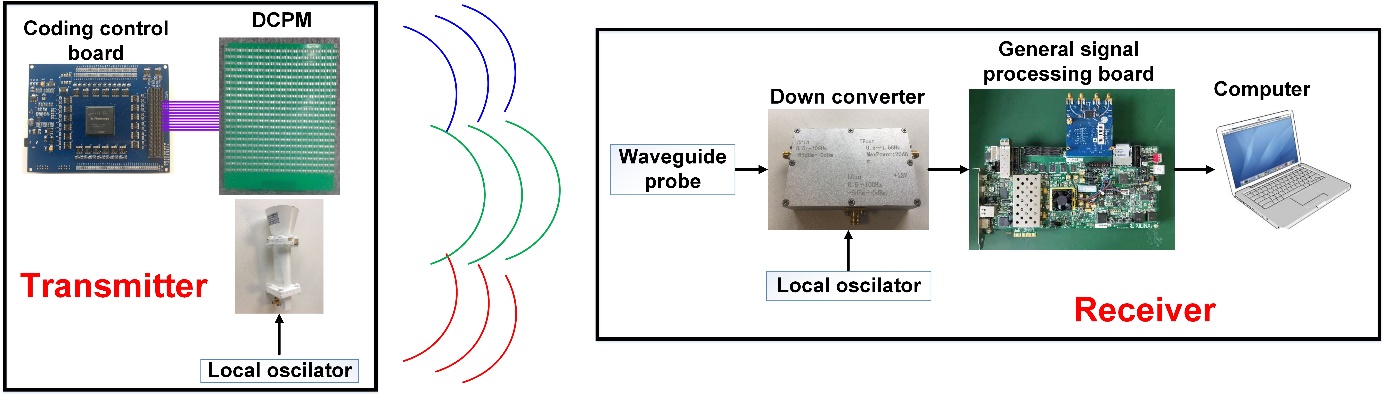


**Fig. S3 Schematic diagram of the multi-channel transmission.** The DCPM is used to transmit information through multiple channels, and the receiver will sample the signals and record the waveforms.

Figure S3 illustrates the schematic diagram of the multi-channel transmission. The coding schemes of DCPM had been stored in the coding control board in advance. A horn antenna was used to illuminate the DCPM sample. By configuring DCPM, the waves radiated from the horn antenna were modulated in space and time domain to transmit different symbols in different channels.

Because the carrier frequency of the signals is 10GHz, the sampling frequency has to be higher than 20GHz according to Nyquist sampling theory. For the convenience of sampling, the carrier frequency of the signals was converted to a much lower value. At first, a down converter was used to convert 10GHz to 1GHz. The general signal processing board converted it from 1GHz to 3MHz, and then the signals were sampled with a sampling frequency of 31.44MHz. The waveforms in the three channels have been displayed in Fig. 3.

**Supplementary Note 4: Geometries and scattering coefficients of the programmable unit**


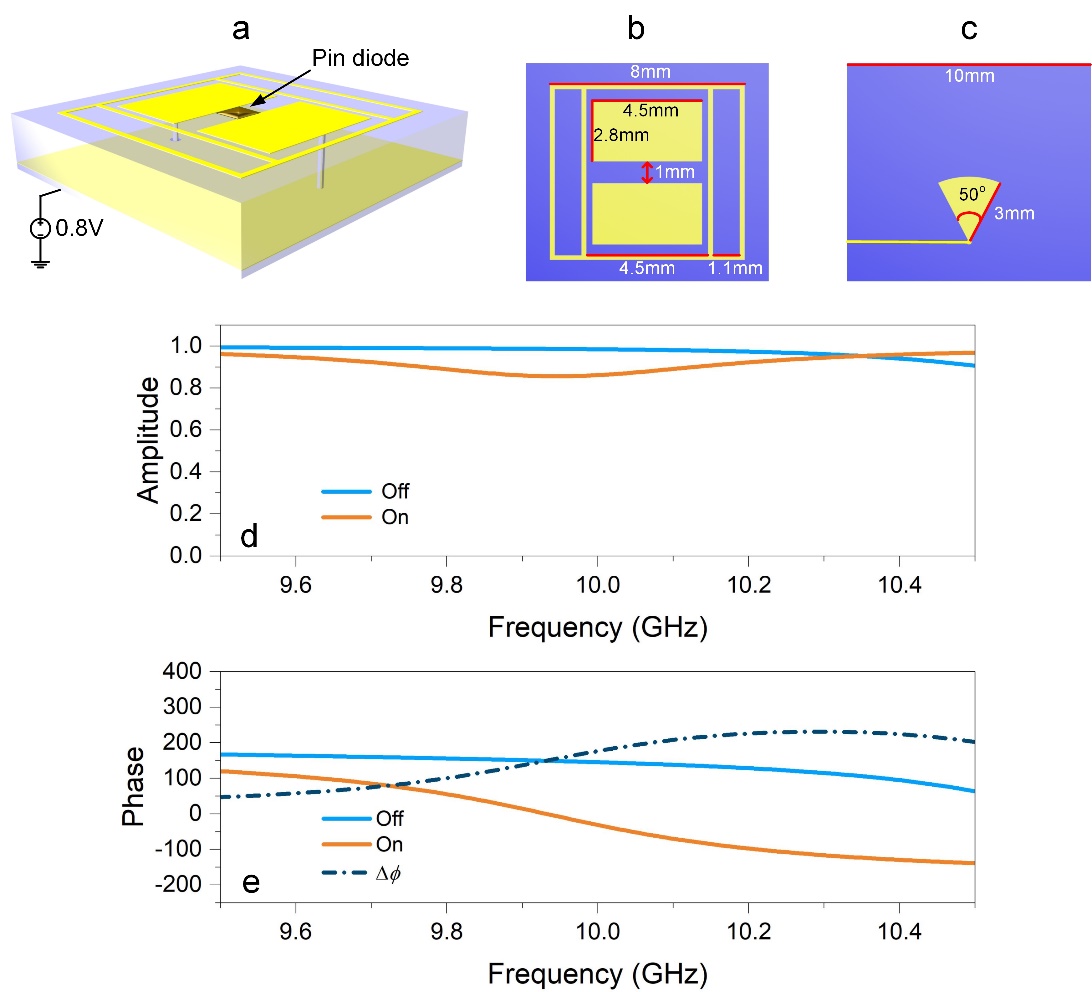


**Fig. S4 Geometries of the digital coding element.** (**a**) Side view, in which the dark patch represents the pin diode with the break-over voltage of 0.8V. (**b**) Bottom view. (**c**) Top view. (**d**) Reflection phases of the element at different PIN states, in which the dashed line gives the phase difference. (**e**) Reflection amplitudes of the element at different PIN states.

In digital coding programmable metasurface (DCPM), the elements are binary encoded. Hence each element of the metasurface can only render two kinds of phases (e.g. opposite phases), amplitudes (e.g. total reflection and transmission), or polarizations (e.g. cross polarizations). Each element contains a pin diode which decides the state the binary element. Here, binary opposite phases are used to construct DCPM. Figure S4 shows the details of the digital coding element, which is composed of three metallic layers and two dielectric layers. The permittivity of the first dielectric layer is 2.65 with height of 1.6mm, while the permittivity of the second dielectric layer is 3 with height of 0.2mm. The top metallic layer is integrated with a pin diode; the middle metallic layer serves as a reflective surface and also as ground of DC signals; while the bottom metallic layer is a fan-shaped structure to choke the high-frequency signals from the DC signals. Two metallic cylinders are used to drive the pin diode, one of which is connected to the middle metallic layer, and the other penetrates the middle metallic layer to load the DC voltages.

The digital coding element is simulated in commercial software (CST Microwave Studio) to obtain the reflection coefficients. In simulations, the pin diode is modeled as an inductance (0.75nH) and a shunt impedance (0.5Ω) when it is turned on, or an inductance (0.5nH) and a series capacitor (0.24pF) when it is turned off. By applying different voltages, the pin diode will be switched on and off, leading to opposite reflection phases and similar reflection amplitudes, as can be observed from the simulated results. Hence, the particle can be treated as a binary phase element. Specifically, the particle is represented by code “0” when the pin diode is switched off, and code “1” when it is switched on. The dashed line in Fig. S4d shows that the opposite phases appear around 10GHz, and hence the binary phase element is defined at 10GHz.

**Supplementary Note 5: An example of near-field imaging**


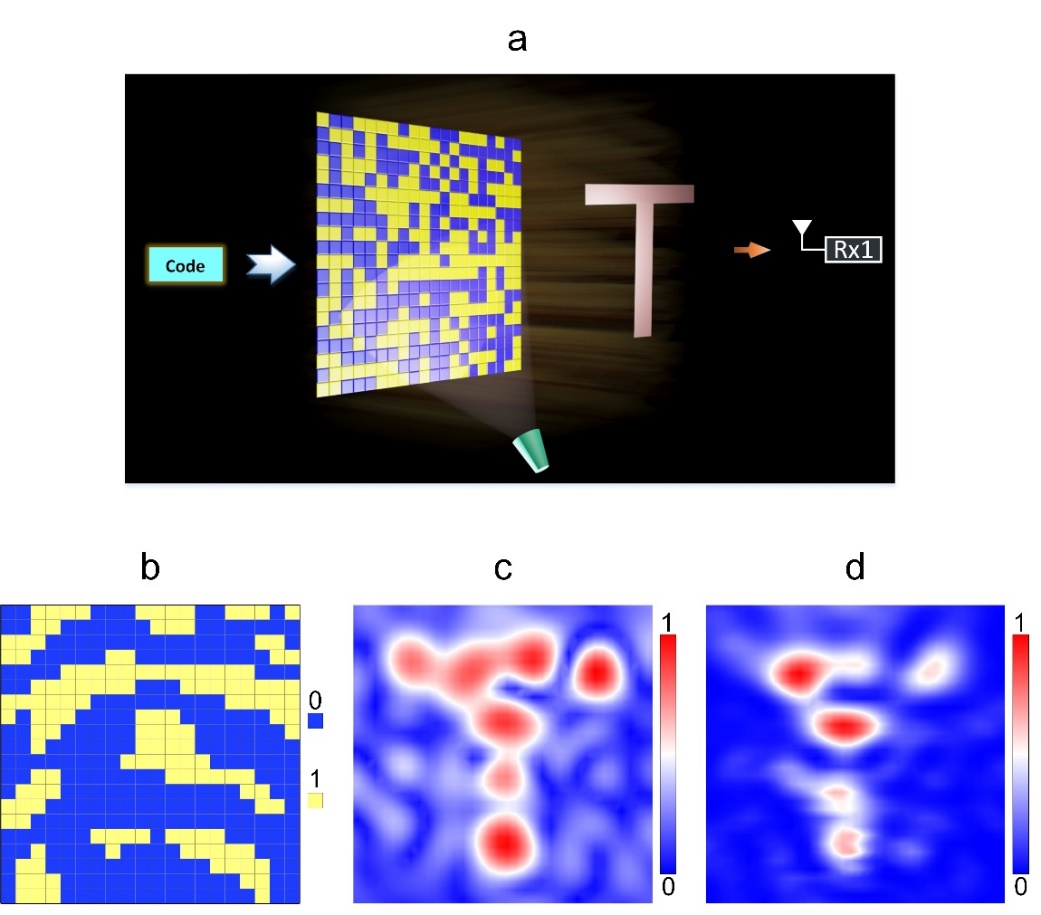


**Fig. S5 Near-field imaging based on a DCPM.** (**a**) Framework of the scheme. (**b**) Synthesized aperture code, in which the size of the coded aperture is 200×200mm^2^. (**c**) Normalized near-field pattern from calculations with the size of 648×648mm^2^. (**d**) Normalized near-field pattern from measurements with the size of 648×648mm^2^.

In Fig. S5, the waves radiated from the horn antenna are modulated by DCPM to construct a character “T” on a near-field plane. By using the modified GS algorithm, the corresponding aperture codes and near-field image are obtained. The fabricated DCPM sample was configured corresponding to the calculated codes, and was measured in a near-field chamber. Figure S5d shows the measured near-field pattern, which is consistent with the calculated results. The distortion is resulted from shielding and scattering effects of the trestle and the feeding horn antenna. Besides, the errors exist inevitably in the modeling of pin diode and in fabrication procedures of DCPM. Increasing the size of DCPM will help to improve the quality of the near-field image. The measured results have proven that DCPM is able to construct near-field images by directly configuring the aperture codes.

**Supplementary Note 6: Derivations of the aperture codes**


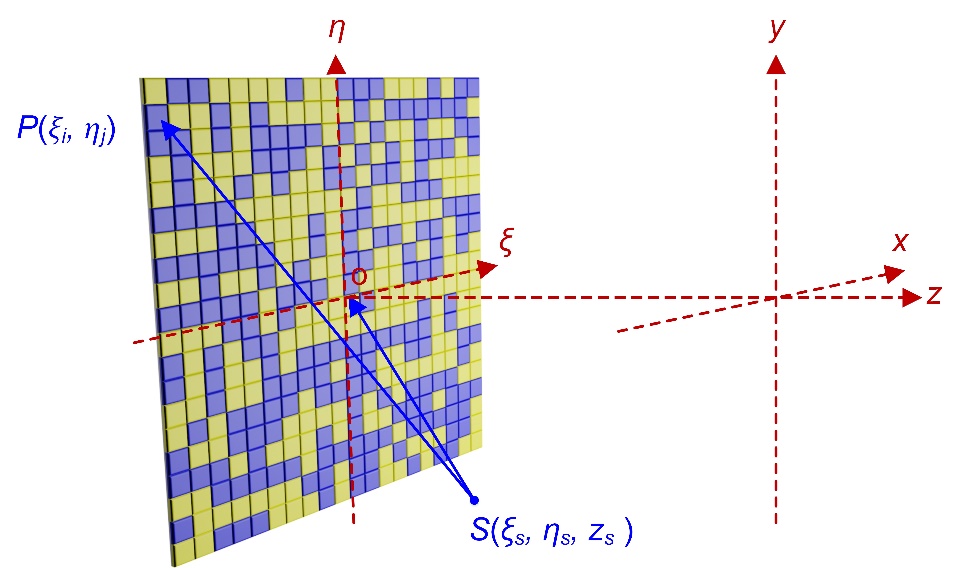


**Fig. S6 Schematic of compensation to the illumination phase.**

As DCPM is illuminated by a near-field source, the radiation phases recovered from a specified near-field pattern will be compensated by the illumination phases, and then be discretized into binary forms. Assuming that the aperture field on DCPM is written as

$U\left( \xi_{i},\eta_{j} \right)=A(\xi_{i},\eta_{j}{)e}^{j\varphi(\xi_{i},\eta_{j})}$ (1)

in which $A(\xi_{i},\eta_{j})$ is the radiation intensity and $\varphi(\xi_{i},\eta_{j})$ is the radiation phase, then the reflection phase of each element is given by: $\varphi_{R}\left( \xi_{i},\eta_{j} \right)=\varphi\left( \xi_{i},\eta_{j} \right)-\varphi_{I}\left( \xi_{i},\eta_{j} \right)$, where $\varphi_{I}\left( \xi_{i},\eta_{j} \right)$ is the illuminating phase. In the present design, the coordinate of the point source S is (0, -170, 275) mm, and hence the illuminating phase of each element on DCPM is expressed as $\varphi_{I}\left( \xi_{i},\eta_{j} \right)=-k\cdot\sqrt{\xi_{i}^{2}+{(\eta_{j}+0.17)}^{2}+{0.275}^{2}}$. When applying the modified GS algorithm, the radiation amplitude $A(\xi_{i},\eta_{j})$ cannot be simply replaced by the reflection amplitude because the feeding source is not an idea plane wave. It is calculated by the following expression:

$A(\xi_{i},\eta_{j})=\frac{\cos\left( \theta_{f} \right)}{r_{f}}\cdot cos(\theta_{e})\cdot\left| \Gamma(\xi_{i},\eta_{j}) \right|$ (2)

in which $\cos\left( \theta_{f} \right)$ is the approximation of the radiation pattern of the horn antenna, and $\theta_{f}$ is the angle between *SO* and *SP*; $cos(\theta_{e})$ is the approximation of the radiation pattern of the binary element, and $\theta_{e}$ is the angle between *SP* and *z* axis; $r_{f}$ is the length of *SP*; and $\Gamma(\xi_{i},\eta_{j})$ is the reflection coefficient of the binary element.

20×20=400 digital coding elements were used to construct the practical DCPM, and all elements are independently controlled by the DC voltages from a customized circuit board. A horn antenna was used to illuminate DCPM. Figure S6 shows the schematic, in which the antenna was represented by a point source located at the phase center of the horn antenna. By configuring digital codes on DCPM, the near field is modulated in space domain. According to Fresnel diffraction theory, the near field can be derived as

$U\left( x,y \right)=\frac{e^{-jkz}}{-j\lambda z}e^{-j\frac{k}{2z}(x^{2}+y^{2})}\iint_{-\infty}^{+\infty} [U\left( \xi,\eta\right)e^{-j\frac{k}{2z}(\xi^{2}+\eta^{2})}]e^{j\frac{2\pi}{\lambda z}(x\xi+y\eta)}d\xi d\eta$, (3)

in which ($\xi,\eta$) and ($x,y$) represent the coordinates of DCPM and imaging plane, respectively; *z* is the distance between the aperture and imaging plane; and $k$ and $\lambda$ are wavenumber and wavelength, respectively. It is observed that, except a phase correction term, the near field is a Fourier transform of the aperture field. Hence, if the near field is specified, the corresponding aperture field can be obtained by using modified Gerchberg-Saxton (GS) algorithm^38^.
